# Light-Mediated Growth of Noble Metal Nanostructures (Au, Ag, Cu, Pt, Pd, Ru, Ir, Rh) From Micro- and Nanoscale ZnO Tetrapodal Backbones

**DOI:** 10.3389/fchem.2018.00411

**Published:** 2018-09-10

**Authors:** Trevor B. Demille, Robert A. Hughes, Arin S. Preston, Rainer Adelung, Yogendra Kumar Mishra, Svetlana Neretina

**Affiliations:** ^1^Department of Aerospace and Mechanical Engineering, College of Engineering, University of Notre Dame, Notre Dame, IN, United States; ^2^Functional Nanomaterials, Institute for Materials Science, Kiel University, Kiel, Germany; ^3^Department of Chemistry and Biochemistry, University of Notre Dame, Notre Dame, IN, United States; ^4^Center for Sustainable Energy at Notre Dame, Notre Dame, IN, United States

**Keywords:** ZnO, tetrapod, light-mediated, catalysis, synthesis, nanoparticle, 4-nitrophenol

## Abstract

Micro- and nanoscale ZnO tetrapods provide an attractive support for metallic nanostructures since they can be inexpensively produced using the flame transport method and nanoparticle synthesis schemes can take advantage of a coupled response facilitated by the formation of a semiconductor-metal interface. Here, we present a light-mediated solution-based growth mode capable of decorating the surface of ZnO tetrapods with nanostructures of gold, silver, copper, platinum, palladium, ruthenium, iridium, and rhodium. It involves two coupled reactions that are driven by the optical excitation of electron-hole pairs in the ZnO semiconductor by ultraviolet photons where the excited electrons are used to reduce aqueous metal ions onto the ZnO tetrapod as excited holes are scavenged from the surface. For the most part, the growth mode gives rise to nanoparticles with a roundish morphology that are uniformly distributed on the tetrapod surface. Larger structures with irregular shapes are, however, obtained for syntheses utilizing aqueous metal nitrates as opposed to chlorides, a result that suggests that the anion plays a role in shape determination. It is also demonstrated that changes to the molarity of the metal ion can influence the nanostructure nucleation rate. The catalytic activity of tetrapods decorated with each of the eight metals is assessed using the reduction of 4-nitrophenol by borohydride as a model reaction where it is shown that those decorated with Pd, Ag, and Rh are the most active.

## Introduction

As a wide bandgap (3.37 eV) semiconductor exhibiting high electron mobility, room temperature luminescence, and piezoelectricity, ZnO has garnered intense interest (Kumar et al., 2017; Laurenti and Valentina, 2017; Chaudhary et al., 2018; Vishnukumar et al., 2018). Its relevance has been further heightened by the ability to synthesize intricate geometries on both the nanoscale and microscale (Janotti and Van de Walle, 2009). A geometry of particular interest is the ZnO tetrapod, which has four arms connected to a central core. Tetrapods are typically formed using a non-catalytic growth mode that sees the nucleation of a zinc blende crystal core from which four wurtzite crystal arms emerge, a growth mode that is common to II–VI semiconductors (Newton and Warburton, 2007). Such structures are of intense interest due to potential applications in electronics, sensing, biomedicine, catalysis, and composites (Bai et al., 2008; Abdulgafour et al., 2012; Castillejos et al., 2012; Papavlassopoulos et al., 2014; Naghizadeh-Alamdari et al., 2015; Picciolini et al., 2015; Alsultany et al., 2016; Gröttrup et al., 2017a; Mishra and Adelung, 2017). With a high Young's modulus, the ability to withstand high temperatures, and the capability to be produced in bulk quantities from earth abundant materials, ZnO tetrapods can also act as inexpensive structural backbones (Newton and Warburton, 2007; Janotti and Van de Walle, 2009; Mecklenburg et al., 2012; Silva et al., 2017). Decorating micro- and nanoscale ZnO tetrapods with metal nanostructures is a particularly intriguing prospect in the respect that the tetrapod provides a retrievable support for use in liquid-phase heterogeneous catalysis while simultaneously providing the potential for a coupled response at the semiconductor-nanometal interface.

ZnO tetrapods decorated with noble metal nanostructures (e.g., Au, Ag, and Pt) and metal-oxides are known to enhance various sensing modalities and optoelectronic responses (Ammari et al., 2004; Zhang et al., 2007; Tan et al., 2008; Wang et al., 2011b; Rackauskas et al., 2015; Bertoni et al., 2016; Sun et al., 2016; Schütt et al., 2017; Wu et al., 2017). In particular, methods for synthesizing Au nanostructures on the ZnO tetrapod surface have been successfully demonstrated through liquid-phase deposition-precipitation (Castillejos et al., 2012), light-mediation (Picciolini et al., 2015), and physical vapor phase techniques (Silva et al., 2017). Coupled with Au nanostructures, ZnO tetrapods have been shown to increase the sensitivity of SERS (surface-enhanced Raman spectroscopy) surfaces (Picciolini et al., 2015). ZnO tetrapods decorated with noble metals have also been investigated as a tool in the assessment of plasma concentrations, food processing, biomolecular sensing, high energy imaging, and radiation detection (Tarrago-Trani et al., 2006; Podila et al., 2011; Castillejos et al., 2012; Picciolini et al., 2015; Sun et al., 2016). Ag-decorated ZnO tetrapods are valued because of the intense plasmonic resonances exhibited by Ag nanoparticles in the visible spectrum. Such structures have been demonstrated as highly efficient photocatalysts in model reactions such as the degradation of methylene blue and methylene orange (Wang et al., 2011a; Li et al., 2013; Naghizadeh-Alamdari et al., 2015; Rackauskas et al., 2015; Bertoni et al., 2016). There have also been demonstrations employing thin film evaporation to produce surface structures and even hybrid synthesis techniques that introduce metallic precursors during the tetrapod assembly stage (Giorgio et al., 1995; Ammari et al., 2004; Fouad et al., 2011; Gröttrup et al., 2017b). These techniques, however, rarely produce high nanostructure number densities, monodisperse metal nanoparticles, or consistent nanostructure geometries.

Solution-based chemistry provides the most versatile route for forming supported noble metal nanostructures on oxide supports (Lee et al., 2014; Li and Tang, 2014; Munnik et al., 2015; Neretina et al., 2016). Such routes can, however, prove challenging when using ZnO tetrapods since the chemical environments used in many of these liquid-phase syntheses attack the ZnO surface. Light-mediated solution-based growth modes present a possible means for mitigating this concern since they can be carried out at room temperature under relatively mild reaction conditions. While light-mediated growth modes have been successfully demonstrated for Au (Castillejos et al., 2012; Picciolini et al., 2015; Bertoni et al., 2016) and Ag (Wang et al., 2011b; Wu et al., 2017), a single-step light-driven synthesis has not yet been demonstrated for decorating ZnO tetrapods with a wide variety of metallic nanostructures. Here, we demonstrate the surface decoration of ZnO tetrapods with Au, Ag, Pt, Cu, Pd, Ir, Rh, and Ru using a liquid-phase UV light-mediated synthetic pathway. The so-formed structures are then assessed as catalysts using the model reaction that sees 4-nitrophenol (4-NP) reduced to 4-aminophenol (4-AP) by borohydride.

## Results and discussion

### Synthesis

**Figure 1:**
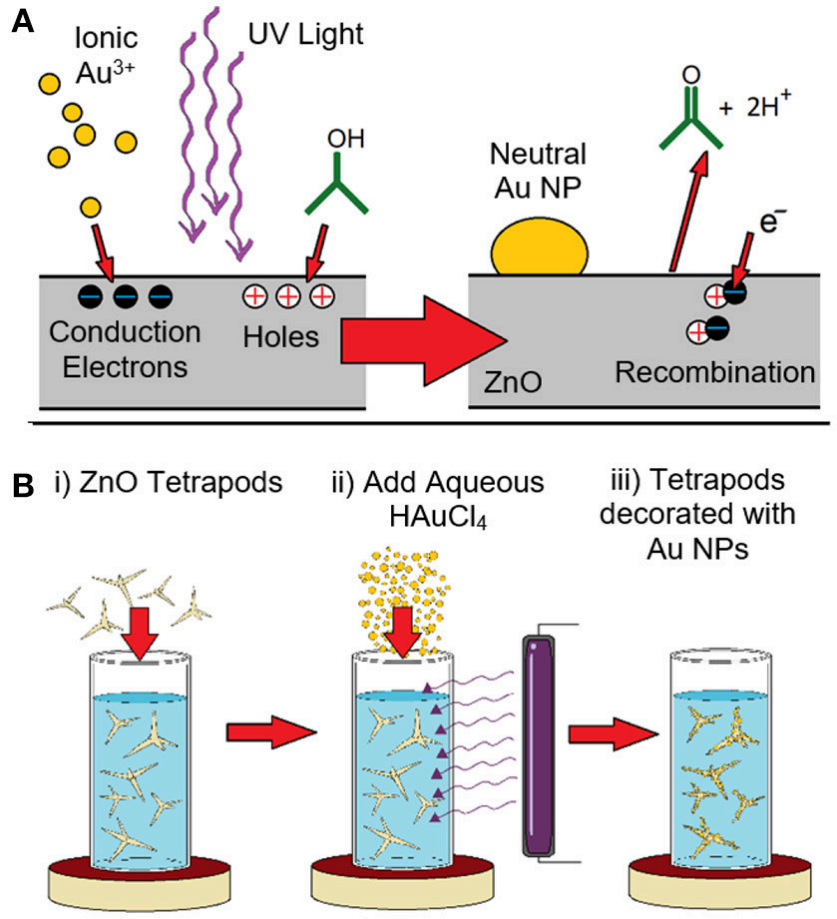
**(A)** Schematic representation of the light-mediated growth mode used to decorate ZnO tetrapods with Au nanoparticles. The incident UV light excites electron-hole pairs in the ZnO. The electrons excited to the conduction band reduce Au^3+^ ions which leads to Au deposition. The holes in the valence band are scavenged through recombination with electrons derived from a reaction which sees the oxidative reduction of isopropyl alcohol to acetone as well as the release of H^+^ ions into the solution. **(B)** Schematic showing the synthesis process in which (i) ZnO tetrapods are added to isopropyl alcohol and stirred, (ii) the reaction is initiated through the addition of aqueous HAuCl_4_ and the application of UV light, and (iii) Au nanoparticles (NPs) form on the ZnO tetrapods.

Figure 1 schematically shows the chemical processes used to generate ZnO tetrapods decorated with metal nanostructures. The synthesis was adapted from a previously reported optically-driven growth mode (Picciolini et al., 2015; Bertoni et al., 2016). It is reliant on ZnO tetrapods being suspended in liquid reactants that support two coupled reactions that are driven by UV photons. The reaction is initiated by the optical excitation of electron-hole pairs in the ZnO semiconductor. With electrons excited above the band gap into the conduction band and Au^3+^ ions in the adjacent solution, the conditions exist for a reaction that sees the ions reduced to Au^0^, a neutral species which deposits on the surface and acts as a favorable nucleation site for continued deposition. The reduction of each Au^3+^ ion leaves behind three holes in the valence band and the overall Au/ZnO structure with a charge of 3^+^. At the same time, the adjacent solution has a corresponding negative charge. Such a charge build-up would ultimately prevent the reduction and deposition of further Au^3+^ ions due to Coulombic repulsion. This situation is, however, remedied by a second reaction that sees the oxidative reduction of isopropyl alcohol to acetone (Ait-Ichou et al., 1984; Yamakata et al., 2002, 2003). In this reaction, electrons are injected into the ZnO while positive H^+^ ions enter the liquid phase. With this reaction giving rise to a charge imbalance that is opposite to the Au^3+^ reduction reaction, the electro-neutrality of both the structure and the solution is preserved. (Picciolini et al., 2015) The various chemical processes occurring at the ZnO surface are illustrated in Figure 1A.

The tetrapods used in the light-mediated growth mode were synthesized using the flame transport method, the details of which can be found elsewhere (Mishra et al., 2013). Briefly, micrometer-scale metallic precursors, sacrificial polymer polyvinyl butyral (PVB), and ethanol in a specific weight ratio are heated in a muffle furnace (open-air) where the oxygen concentration required for ZnO tetrapod growth is tuned by adjusting the amount O_2_ consumed in the combustion of PVB and ethanol. Decreasing the concentration of these reactants, hence, results in greater quantities of oxygen available for the ZnO synthesis. As the reactants are transported through the furnace by convection, ZnO nanoparticles first nucleate and, with time, evolve into tetrapods with micro and nanoscale dimensions. The tetrapods are then collected on substrates or from the inside of the furnace. The as-synthesized tetrapods are then readied for nanoparticle decoration by suspending them in isopropyl alcohol that is rapidly stirred to inhibit their precipitation. The reaction is initiated by exposing the solution to 4 W 365 nm UV light as a small quantity of aqueous metal ions is injected. In addition to the ZnO tetrapod preparation method, the use of a low wattage UV light source marks a key difference between this work and the prior study (Bertoni et al., 2016) which utilized a broadband 300 W halogen lamp. Metal nanostructure growth is allowed to proceed over a 2 h interval where the metal ion supply is periodically replenished though additional injections. The reaction is terminated by turning off the UV light source. In the absence of stirring, the metal decorated ZnO tetrapods readily precipitate and are easily harvested. These structures are then cleaned and re-suspended in fresh isopropyl alcohol, and stored as such. The synthesis procedure is schematically depicted in Figure 1B.

### Au nanoparticle decoration

**Figure 2:**
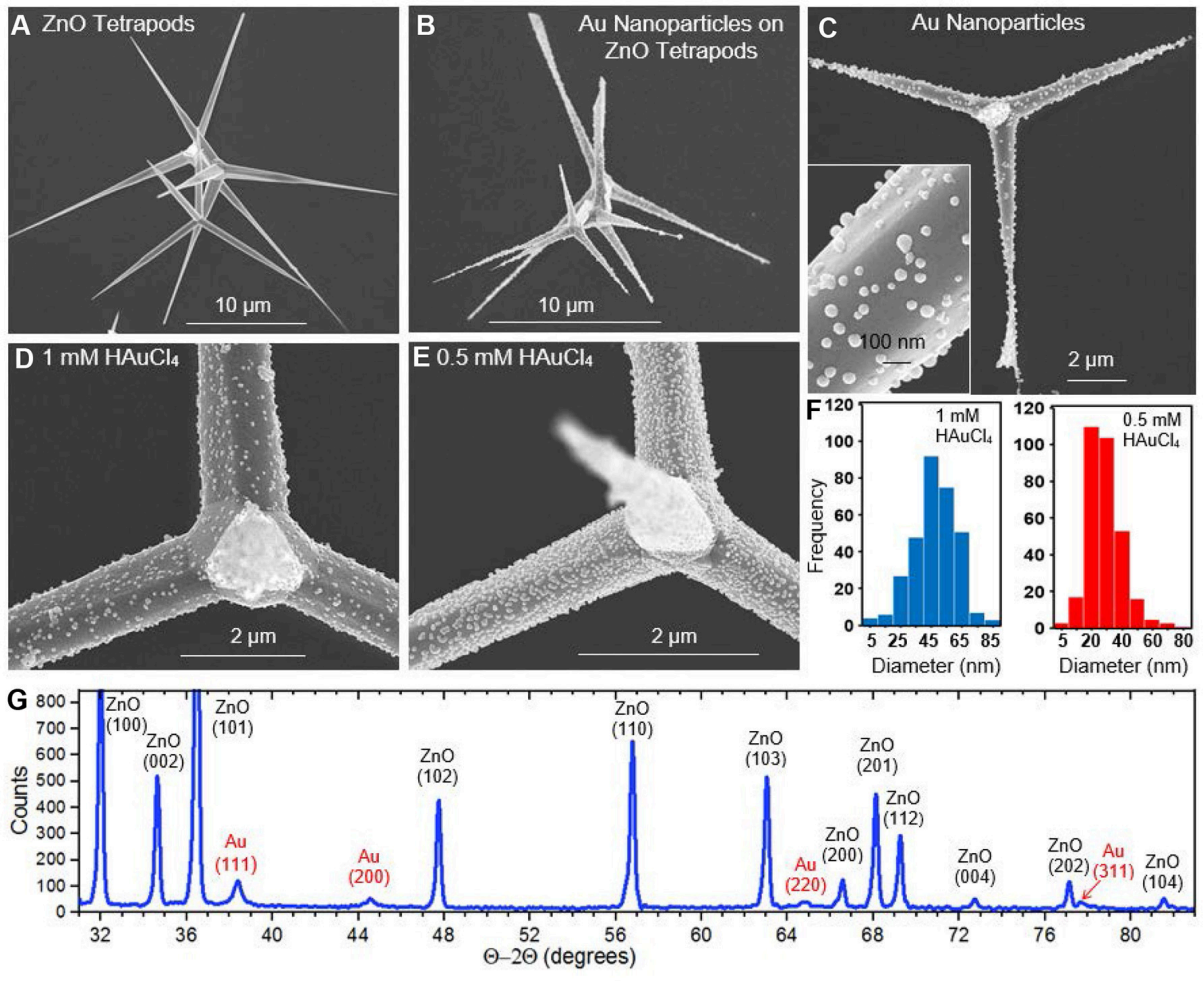
SEM images showing **(A)** bare ZnO tetrapods **(B–D)**, Au nanoparticles synthesized on ZnO tetrapods from 1 mM HAuCl_4_ **(E)**, Au nanoparticles synthesized on ZnO tetrapod from 0.5 mM HAuCl_4_ **(F)**, histograms showing the Au nanoparticle size distribution derived from 1 mM (blue) and 0.5 mM (red) HAuCl_4_. **(G)** XRD data showing the Au and ZnO reflections.

Figure 2A shows an SEM image of ZnO tetrapods produced using the flame transport method. The structures, which vary widely in size, have tapered arms that can extend tens of micrometers in length and exhibit a solid or hollow interior. Figures 2B–E shows a series of SEM images of Au-decorated tetrapods derived from the light-mediated synthesis. For all cases, the ZnO tetrapods appear structurally intact, showing no indication of any chemical attack due to the light-mediated synthesis. The Au nanostructures that decorate the ZnO surface appear as unfaceted roundish nanoparticles. The separation and size of the nanostructures is dependent on the molarity of HAuCl_4_ used. Figures 2D–E compares ZnO tetrapods decorated with Au nanoparticles derived from HAuCl_4_ molarities of 1 and 0.5 mM, respectively. The lower molarity gives rise to a more densely decorated ZnO surface, a result that is highly reproducible. Histograms of the size distribution for the two cases (Figure 2F) indicate mean nanoparticle diameters of 47.5 and 31.8 nm for the 1 and 0.5 mM concentrations where, in both cases, the standard deviation is ~14 nm. At the lower concentration, the number density of Au^3+^ ions available at the ZnO tetrapod surface is a factor of two less. The number of available conduction band electrons, however, is the same since the incident light intensity is maintained at the same value for all syntheses. The result indicates that, under these conditions, a lower Au^3+^ concentration in the adjacent solution provides a more favorable condition for the nucleation of additional Au nanoparticles as opposed to continued deposition onto preexisting structures. While the higher density of nanoparticles gives the impression that more Au is deposited on the ZnO tetrapods for 0.5 mM concentration, this is not the case. An analysis of the volume of Au deposited per unit area over the course of the 2 h synthesis indicates that 1.7 × more Au is deposited when using the 1 mM concentration. This estimate was made by analyzing the size distribution over representative areas imaged in SEM from which the total volume of Au was calculated. XRD characterization of the Au-decorated ZnO tetrapods using the Bragg-Brentano Θ−2Θ configuration is shown in Figure 2G. With all reflections being attributed to ZnO or Au, the result confirms that no unexpected phases originate from the flame transport method or the light-mediated growth mode.

### Nanoparticle decoration with Pt-group metals

**Figure 3:**
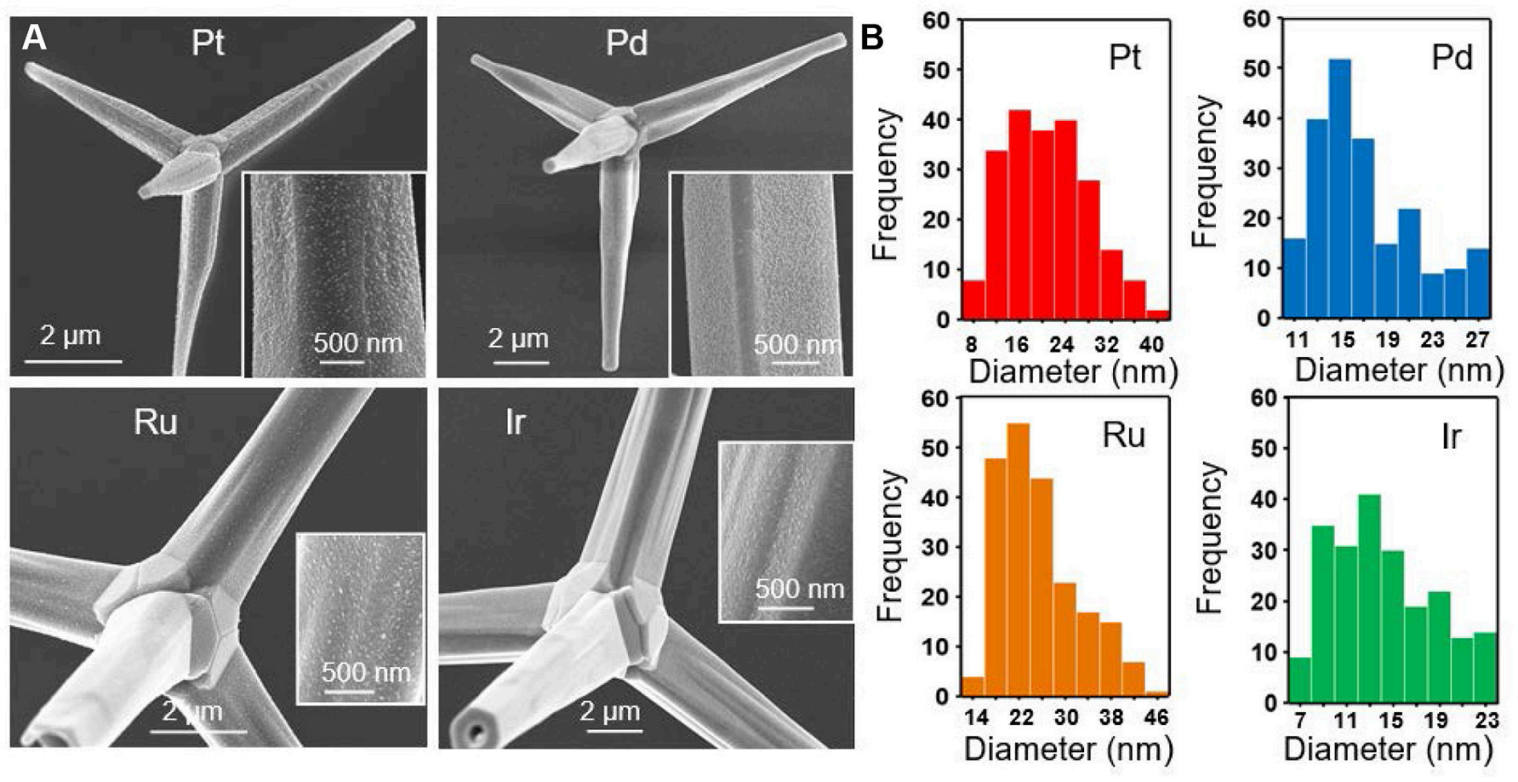
**(A)** SEM images of ZnO tetrapods decorated with Pt, Pd, Ru, and Ir nanoparticles and **(B)** their corresponding size distribution histograms.

Light-mediated syntheses of Pt-group metals were carried out in an analogous manner using aqueous K_2_PtCl_4_, Na_2_PdCl_4_, RuCl_3_, H_2_IrCl_6_, and Na_3_RhCl_6_, to obtain Pt-, Pd-, Ru-, Ir-, and Rh-decorated tetrapods, respectively. Figure 3 shows SEM images of the Pt-, Pd-, Ru-, and Ir-decorated tetrapods and their corresponding size distributions. For all cases, the nanoparticles have a roundish geometry similar to that observed for Au. The structures are, however, smaller in size, more densely spaced, show greater monodispersity, and grow at a slower rate. For the Pt, Pd, Ru, and Ir nanoparticles the size distribution histograms indicate mean particle diameters of 20.91, 17.29, 25.34, and 14.12 nm with standard deviations of 7.03, 4.67, 6.99, and 4.68 nm, respectively. These characteristically smaller and more monodisperse nanoparticles form at a high density which indicates a greater propensity for the reduced metallic ions to nucleate at a new site on the ZnO tetrapod surface as opposed to their deposition onto pre-existing nanoparticles—an effect similarly seen for Au nanoparticle syntheses employing lower HAuCl_4_ concentrations (Figure 2E). The Ir synthesis is unique in that nanostructures preferentially nucleate on the tetropod arms as opposed to the core (Supplementary Figure 1). The Rh synthesis was also unique in that it was difficult to resolve a significant number of nanoparticles on the tertrapod surface with SEM. A small Rh signature was, however, obtained using Energy-dispersive X-ray spectroscopy (EDS). Moreover, the Rh-decorated tetrapods showed significant catalytic activity (*vida infra*). Together these results suggest that Rh nanoparticles are present on the tetrapod in numbers of significance, but where their size is below the resolution limit of the SEM. Characterization of the Rh-decorated tetrapods is presented as Supplementary Material (Supplementary Figure 2) along with EDS data for the other metals (Supplementary Figure 3).

### Nanoparticle decoration with Cu and Ag

**Figure 4:**
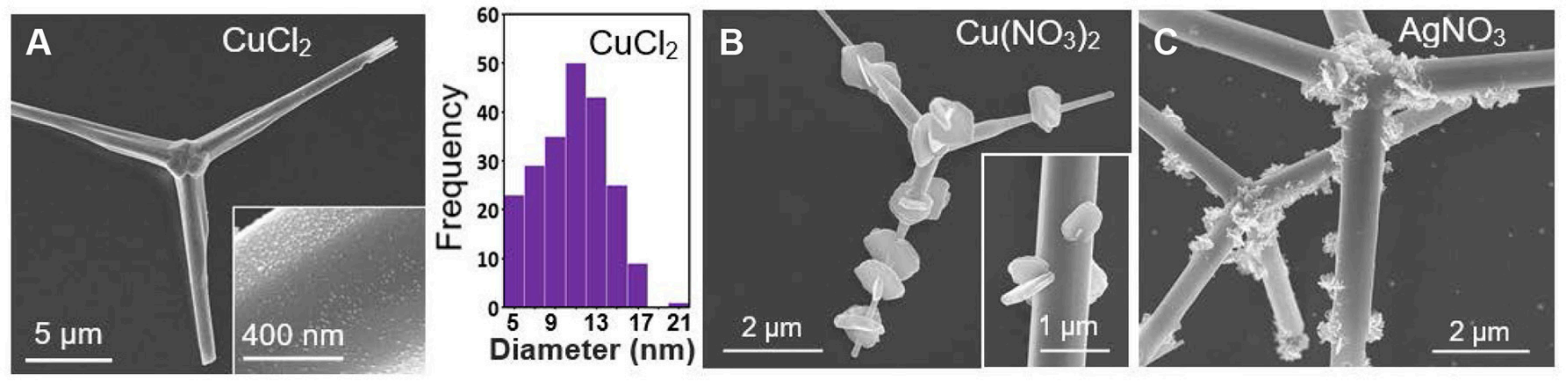
**(A)** SEM images of Cu-decorated ZnO tetrapods derived from aqueous CuCl_2_ and the corresponding nanoparticle size distribution histogram. SEM images of **(B)** Cu- and **(C)** Ag-decorated ZnO tetrapods derived from aqueous Cu(NO_3_)_2_ and AgNO_3_, respectively.

Aqueous solutions of CuCl_2_ and Cu(NO_3_)_2_ were both investigated as a means to decorate ZnO tetrapods with Cu nanoparticles. The CuCl_2_ results, shown in Figure 4A, were largely in line with those obtained for other metals in that it yielded a rounded nanoparticle geometry with an average diameter of 11.53 nm. In stark contrast, Cu(NO_3_)_2_ yielded a distinct morphology characterized by micrometer-scale plates that extend radially outward from the arms of the ZnO tetrapod (Figure 4B). It is noted that Wei et al. (2014) observed markedly different morphologies when depositing Cu nanostructures on ZnO nanowires using the same two salts, yielding small nanoparticles for CuCl_2_ and leaf-shaped structures for Cu(NO_3_)_2_. The growth of large plate-like structures off preexisting structures is not unprecedented. It has, for example, been observed for both Cu and Ag when deposited on Au templates (Gilroy et al., 2014; Mettela and Kulkarni, 2015; Zhu et al., 2018). Markedly different morphologies are also observed when the light-mediated growth mode is used to decorate the tetrapods with Ag using aqueous AgNO_3_. For this case, Ag structures form with a jagged shape (Figure 4C). It is conceivable that the ${\text{NO}}_{3}^{-}$ counterion derived from Cu(NO_3_)_2_ and AgNO_3_ is crucial to the formation of these anomalously shaped structures since all the chloride-based salts used in this study give rise to small rounded nanostructures.

### Catalysis

The catalytic reduction of 4-NP is widely recognized as a trusted model reaction for gauging the catalytic efficacy of nanoscale materials (Hervés et al., 2012; Aditya et al., 2015; Zhao et al., 2015). In this reaction, aqueous 4-NP is reduced to 4-AP by borohydride on the surface of a catalyst. A 4-NP absorbance peak at 400 nm is used to spectroscopically monitor the progress of the reaction in real-time. In the presence of a catalyst, the time dependence of the 400 nm absorbance takes the form of an exponential decay. An apparent reaction rate constant, k_app_, can be extracted from the data using the expression:

(1) $$\begin{array}{l} \ln\left(\frac{A}{A_{0}}\right)=\;k_{app}t \end{array}$$

where A/A_0_ is the absorbance at time t normalized to its initial value. A plot of the natural logarithm of the normalized absorbance vs. time, therefore, yields a straight line with a slope of k_app_ where larger values indicate a better performing catalyst. When assessing catalytic performance, it is imperative that the dissolved oxygen be purged from all aqueous reactants since its presence (i) gives rise to an induction time (Menumerov et al., 2016), (ii) diminishes k_app_ values (Menumerov et al., 2017), (iii) can result in the oxidative etching of catalytically active sites (Menumerov et al., 2018), and (iv) lead to the misidentification of the true catalyst (Menumerov et al., 2018).

**Figure 5:**
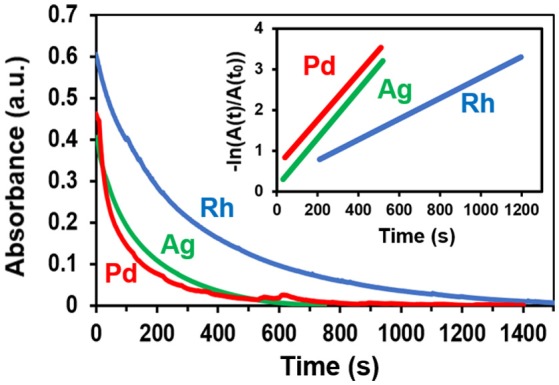
The time-dependence of the 400 nm 4-NP absorbance when using Rh-, Ag-, and Pd-decorated tetrapods as catalysts. The inset depicts the linear region of the normalized logarithmic plot of the absorbance from which the reaction rate is extracted.

The catalytic activity of ZnO tetrapods decorated with metal nanostructures were assessed using solutions of 30 μM 4-NP and 3 mM NaBH_4._ Bare ZnO tetrapods (i.e., those without metallic decoration) exhibited no catalytic activity toward the reduction of 4-NP. Of the eight different metals tested, significant catalytic activity was observed for ZnO tetrapods decorated with Pd, Ag, and Rh. Figure 5 shows the time dependent absorbance obtained for these metals as well as the plot used to extract k_app_. The k_app_ values determined for ZnO tetrapod decorated with Ag, Pd, and Rh are 5.9 × 10^−3^ s^−1^, 5.7 × 10^−3^ s^−1^, and 2.5 × 10^−3^ s^−1^, respectively. While Ag and Pd are well-known for their catalytic activity toward the reduction of 4-nitrophenol, Rh has received little attention. Sub 10-nm nanoparticles of Ag are particularly catalytic due to a high density of low coordination surface sites (Menumerov et al., 2016). The catalytic activity of the comparatively large Ag structures shown in Figure 4C is likely attributable to their jagged shape since such a morphology is characterized by surfaces with numerous steps, kinks, and terraces that inevitably express numerous undercoordinated sites from which the catalytic activity is likely derived (Menumerov et al., 2018).

## Conclusion

In summary, we have demonstrated a light-mediated growth mode for decorating ZnO tetrapods with nanostructures of eight different elemental metals. By employing a room temperature reaction under relatively mild reaction conditions, we have avoided pitfalls associated with practicing common solution-based protocols that show chemical incompatibility with the ZnO surface. This study has, hence, forwarded the use of ZnO tetrapods as a support for metal nanostructures, with the potential to motivate new light-mediated synthesis schemes and provide a new platform for advancing applications in sensing, optoelectronic, catalysis, and photocatalysis that are reliant on a coupled response at the semiconductor-nanometal interface.

## Materials and methods

### Chemicals

Solution-based syntheses were carried out using 99.99% hydrogen tetrachloroaurate(III) trihydrate (Alfa Aesar), 99.9999% silver nitrate (Sigma-Aldrich), 99.99% copper (II) nitrate trihydrate (Sigma-Aldrich), 99.99% potassium tetrachloroplatinate(II) (Sigma-Aldrich), 99.999% sodium tetrachloropalladate(II) (Sigma-Aldrich), 99.98% ruthenium(III) chloride hydrate (Sigma-Aldrich), 99.98% hydrogen hexachloroiridate(IV) hydrate (Sigma-Aldrich), 99.98% sodium hexachlororhodate(III) (Sigma-Aldrich), and 100% isopropyl alcohol (Honeywell). Solutions for catalysis were prepared using 4-NP (Fluka), NaBH_4_ (Fluka), and DI water with a resistivity of 18.2 MΩ cm^−^^1^. All chemicals were used as received.

### Tetrapod synthesis

ZnO tetrapods were prepared in bulk by the flame transport synthesis method (Mishra et al., 2013). Micrometer-scale (typically 3–10 μm) Zn precursor particles, polymer polyvinyl butyral, and ethanol are heated in a muffle-type furnace, in air, above 800°C at a rate >100°C min^−1^. The heated atmosphere within the furnace results in laminar convection that is utilized for the transportation and oxidation of the metallic particles and provides the emerging tetrapods with enough local space for uninterrupted growth as they continue to transit upward through the furnace. The size and geometry of the ZnO structure is dependent on the time spent in transit as well as the component material ratios and oven temperature, granting tunablility to the procedure. Once the reaction is complete, the ZnO tetrapods are collected from the inside the furnace.

### Light-mediated growth modes

Ten milligrams of ZnO tetrapods were weighed and added to a 50 mL polypropylene tube into which 48.8 mL of isopropyl alcohol was added and stirred at 680 rpm using a magnetic stirrer. A 100 μL of aqueous 1 mM metallic salt was added every 10 min for 2 h (for a total of 1.2 mL) while under a 365 nm, 4 W mercury vapor lamp. After 2 h the tetrapods were allowed to precipitate out of solution, where they were then rinsed twice with isopropyl alcohol. For SEM imaging, the decorated ZnO tetrapods were then dropcast onto sapphire heated to 90°C.

### 4-nitrophenol reduction

The catalytic reduction of 4-NP to 4-AP was carried out in a 3 mL quartz cuvette with a 1 cm path length and monitored using a UV-Vis spectrometer. The metal decorated ZnO tetrapods were first dried and then 2 mg were added to the empty cuvette. A 2 mL aqueous solution of 3 mM NaBH_4_ and 30 μM 4-NP was then prepared and purged with N_2_ gas for 10 min to remove dissolved oxygen. The cuvette was then filled with the purged solution and covered with Parafilm such that two small openings allowed for N_2_ gas to be flowed over the solution (Menumerov et al., 2016) to further prevent oxygen from dissolving into the reactants during data collection. The time dependent absorbance was measured at 400 nm for metal-decorated tetrapods investigated.

## Instrumentation

SEM and EDS measurements were carried out using a Magellan 400 FEI field emission scanning electron microscope. XRD scans were obtained using a Bruker D8 Advance Davinci diffractometer using Cu Kα1 radiation. Absorbance spectra were acquired with a Jasco V-730 UV-Vis Spectrophotometer. The nanoparticle size distributions presented in Figures 2F, 3B, 4A were processed with ImageJ software using 312, 215, and 215 nanoparticles, respectively.

## Author contributions

TD, RH, and SN designed the experiments and prepared the manuscript. RA and YM performed tetrapod synthesis. TD performed the light-mediated growth syntheses and catalysis experiments. AP carried out XRD characterization.

### Conflict of interest statement

The authors declare that the research was conducted in the absence of any commercial or financial relationships that could be construed as a potential conflict of interest.

## References

[B1] Abdulgafour H. Hassan Z. Ahmed N. Yam F. (2012). Comparative study of ultraviolet detectors based on zno nanostructures grown on different substrates. J. Appl. Phys. 112:074510 10.1063/1.4757619

[B2] Aditya T. Pal A. Pal T. (2015). Nitroarene reduction: a trusted model reaction to test nanoparticle catalysts. Chem. Commun. 51, 9410–9431. 10.1039/C5CC01131K 25872865

[B3] Ait-Ichou A. Formenti M. Pommier B. Teichner S. J. (1984). Photocatalytic dehydrogenation of lsopropanol on Pt/Ti02 catalysts. J. Catal. 91, 293–307. 10.1016/0021-9517(85)90343-4

[B4] Alsultany F. Hassan Z. Ahmed N. (2016). Low-Power UV photodetection characteristics of zno tetrapods grown on catalyst-free glass substrate. *Sensors Actuat*. A Phys. 250, 187–194. 10.1016/j.sna.2016.09.039

[B5] Ammari F. Lamotte J. Touroude R. (2004). An emergent catalytic material: Pt/ZnO catalyst for selective hydrogenation of crotonaldehyde. J. Catal. 221, 32–42. 10.1016/S0021-9517(03)00290-2

[B6] Bai Z. Xie C. Hu M. Zhang S. Zeng D. (2008). Effect of humidity on the gas sensing property of the tetrapod-shaped Zno nanopowder sensor. Mat. Sci. Eng. B 149, 12–17. 10.1016/j.mseb.2007.11.020

[B7] Bertoni G. Fabbri F. Villani M. Lazzarini L. Turner S. Van Tendeloo G.. (2016). Nanoscale mapping of plasmon and exciton in zno tetrapods coupled with au nanoparticles. Sci. Rep. 6:19168. 10.1038/srep19168 26754789 PMC4709633

[B8] Castillejos E. Gallegos-Suarez E. Bachiller-Baeza B. Bacsa R. Serp P. Guerrero-Ruiz A. (2012). Deposition of gold nanoparticles on ZnO and their catalytic activity for hydrogenation applications. Catal. Commun. 22, 79–82. 10.1016/j.catcom.2012.02.016

[B9] Chaudhary S. Umar A. Bhasin K. K. Baskoutas K. (2018). Chemical sensing applications of ZnO nanomaterials. Materials 11:287. 10.3390/ma11020287 29439528 PMC5848984

[B10] Fouad O. A. El Rahman A. Khder S. Dai Q. El-Shall M. S. (2011). Structural and catalytic properties of ZnO and Al_2_O_3_ nanostructures loaded with metal Nanoparticles. J. Nanopart. Res. 13, 7075–7083. 10.1007/s11051-011-0620-8

[B11] Gilroy K. D. Hughes R. A. Neretina S. (2014). Kinetically controlled nucleation of silver on surfactant-free gold seeds. J. Am. Chem. Soc. 136, 15337–15345. 10.1021/ja5081635 25286025

[B12] Giorgio S. Henry C. Chapon C. (1995). HRTEM studies of the epitaxial growth of Pd particles (1-6 Nm) on ZnO micro-prisms. *Microsc. Microanal*. Microstruct. 6, 237–248.

[B13] Gröttrup J. Postica V. Smazna D. Hoppe M. Kaidas V. Mishra Y. K. (2017a). UV Detection properties of hybrid ZnO tetrapod 3-D networks. Vacuum 146, 492–500. 10.1016/j.vacuum.2017.03.017

[B14] Gröttrup J. Schutt F. Smazna D. Lupan O. Adelung R. Mishra Y. K. (2017b). Porous ceramics based on hybrid inorganic tetrapodal networks for efficient photocatalysis and water purification. Ceram. Int. 43, 14915–14922. 10.1016/j.ceramint.2017.08.008

[B15] Hervés P. Pérez-Lorenzo M. Liz-Marzán L. M. Dzubiella J. Lu Y. Ballauff M. (2012). Catalysis by metallic nanoparticles in aqueous solution: model reactions. Chem. Soc. Rev. 41, 5577–5587. 10.1039/C2CS35029G 22648281

[B16] Janotti A. Van de Walle C. G. (2009). Fundamentals of zinc oxide as a semiconductor. Rep. Prog. Phys. 72:126501 10.1088/0034-4885/72/12/126501

[B17] Kumar R. Umar A. Kumar G. Nalwa H. (2017). Antimicrobial Properties of ZnO Nanomaterials: a review. Ceram. Int. 43, 3940–3961. 10.1016/j.ceramint.2016.12.062

[B18] Laurenti M. Valentina C. (2017). ZnO nanostructures for tissue engineering applications. Nanomaterials 7:374. 10.3390/nano7110374 29113133 PMC5707591

[B19] Lee J. Kim S. M. Lee I. S. (2014). Functionalization of hollow nanoparticles for nanoreactor applications. Nano Today 9, 631–667. 10.1016/j.nantod.2014.09.003

[B20] Li G. Tang Z. (2014). Noble Metal Nanoparticle@Metal Oxide Core/Yolk–shell nanostructures as catalysts: recent progress and perspective. Nanoscale 6, 3995–4011. 10.1039/C3NR06787D 24622876

[B21] Li W. Hua F. Yue J. Li J. (2013). Ag@AgCl Plasmon-induced sensitized zno particle for high-efficiency photocatalytic property under visible light. Appl. Surf. Sci. 285, 490–497. 10.1016/j.apsusc.2013.08.082

[B22] Mecklenburg M. Schuchardt A. Mishra Y. K. Kaps S. Adelung R. Lotnyk A.. (2012). Aerographite: ultra lightweight, flexible nanowall, carbon microtube material with outstanding mechanical performance. Adv. Mater. 24, 3486–3490. 10.1002/adma.201200491 22688858

[B23] Menumerov E. Hughes R. A. Golze S. Neal R. Demille T. Campanaro J. (2018). Identifying the true catalyst in the reduction of 4-nitrophenol: a case study showing the effect of leaching and oxidative etching using ag catalysts. ACS Catal. 8, 8879–8888. 10.1021/acscatal.8b02325

[B24] Menumerov E. Hughes R. A. Neretina S. (2016). Catalytic Reduction of 4-nitrophenol: a quantitative assessment of the role of dissolved oxygen in determining the induction time. Nano Lett. 16, 7791–7797. 10.1021/acs.nanolett.6b03991 27960449

[B25] Menumerov E. Hughes R. A. Neretina S. (2017). One-step catalytic reduction of 4-nitrophenol through the direct injection of metal salts into oxygen-depleted reactants. Catal. Sci. Technol. 7, 1460–1464. 10.1039/C7CY00260B

[B26] Mettela G. Kulkarni G. U. (2015). Site selective cu deposition on au microcrystallites: corners, edges versus planar surfaces. Cryst. Eng. Commun. 17, 9459–9465. 10.1039/C5CE01574J

[B27] Mishra Y. K. Adelung R. (2017). ZnO tetrapod materials for functional applications. Mater. Today 21, 631–651. 10.1016/j.mattod.2017.11.003

[B28] Mishra Y. K. Kaps S. Schuchardt A. Paulowicz I. Jin X. Gedamu D. (2013). Fabrication of macroscopically flexible and highly porous 3D semiconductor networks from interpenetrating nanostructures by a simple flame transport approach. Part. Part. Syst. Charact. 30, 775–783. 10.1002/ppsc.201300197

[B29] Munnik P. de Jongh P. E. de Jong K. P. (2015). Recent developments in the synthesis of supported catalysts. Chem. Rev. 115, 6687–6718. 10.1021/cr500486u 26088402

[B30] Naghizadeh-Alamdari S. Habibi-Yangjeh A. Pirhashemi M. (2015). One-pot ultrasonic-assisted method for preparation of Ag/AgCl Sensitized ZnO nanostructures as visible-light-driven photocatalysts. Solid State Sci. 40, 111–120. 10.1016/j.solidstatesciences.2015.01.007

[B31] Neretina S. Hughes R. A. Gilroy K. D. Hajfathalian M. (2016). Noble metal nanostructure synthesis at the liquid-substrate interface: new structures, new insights and new possibilities. Acc. Chem. Res. 49, 2243–2250. 10.1021/acs.accounts.6b00393 27622782

[B32] Newton M. C. Warburton P. A. (2007). ZnO tetrapod nanocrystals. Mater. Today 10, 50–54. 10.1016/S1369-7021(07)70079-2

[B33] Papavlassopoulos H. Mishra Y. K. Kaps S. Paulowicz I. Abdelaziz R. Elbahry M.. (2014). Toxicity of functional nano-micro zinc oxide tetrapods: impact of cell culture conditions, cellular age and material properties. PLoS ONE 9:e84983. 10.1371/journal.pone.0084983 24454775 PMC3890288

[B34] Picciolini S. Castagnetti N. Vanna R. Mehn D. Bedoni M. Gramatica F. (2015). Branched gold nanoparticles on ZnO 3D architecture as biomedical SERS sensors. RSC Adv. 5, 93644–93651. 10.1039/C5RA13280K

[B35] Podila R. Chen P. Reppert J. Rao A. Ke P. (2011). Biomolecular sensing using gold nanoparticle-coated ZnO nanotetrapods. J. Mater. Res. 26, 2328–2333. 10.1557/jmr.2011.147

[B36] Rackauskas S. Klimova O. Jiang H. Nikitenko A. Chernenko K. Shandakov S. (2015). A novel method for continuous synthesis of Zno tetrapods. J. Phys. Chem. C 119, 1345–1352. 10.1021/acs.jpcc.5b03702

[B37] Schütt F. Signetti S. Krüger H. Röder S. Smazna D. Kaps S. (2017). Hierarchal self-entangled carbon nanotube tube networks. Nat. Commun. 8:1215 10.1038/s41467-017-01324-7 29084950 PMC5662747

[B38] Silva E. L. Mishra Y. K. Fernandes A. J. S. Silva R. F. Strobel J. Kienle L. (2017). Direct synthesis of electrowettable carbon nanowall-diamond hybrid materials from sacrificial ceramic templates using HFCVD. Adv. Mater. Int. 4:1700019 10.1002/admi.201700019

[B39] Sun C. Fu Y. Wang Q. Xing L. Liu B. Xue X. (2016). Ultrafast Piezo-photocatalytic degradation of organic pollutions by Ag_2_O/Tetrapod-ZnO nanostructures under ultrasonic/UV exposure. RSC Adv. 6, 87446–87453. 10.1039/C6RA13464E

[B40] Tan T. Li Y. Liu Y. Wang B. Song X. Li E. (2008). Two-step preparation of Ag/tetrapod-like ZnO with photocatalytic activity by thermal evaporation and sputtering. Mater. Chem. Phys. 111, 305–308. 10.1016/j.matchemphys.2008.04.013

[B41] Tarrago-Trani M. Phillips K. Lemar L. Holden J. (2006). New and existing oils and fats used in products with reduced trans-fatty acid Content. J. Am. Diet. Assoc. 106, 867–880. 10.1016/j.jada.2006.03.010 16720128

[B42] Vishnukumar P. Vivekanandhan S. Misha M. Mohanty A. K. (2018). Recent advances and emerging opportunities in phytochemical synthesis of zno nanostructures. Mat. Sci. Semicond. Process. 80, 143–161. 10.1016/j.mssp.2018.01.026

[B43] Wang J. Fan X. M. Tian K. Zhou Z. W. Wang Y. (2011a). Largely Improved photocatalytic properties of Ag/Tetrapod-like ZnO nanocompounds prepared with different peg contents. Appl. Surf. Sci. 257, 7763–7770. 10.1016/j.apsusc.2011.04.026

[B44] Wang J. Fan X. M. Zhou Z. W. Tian K. (2011b). Preparation of Ag Nanoparticles coated tetrapod-like ZnO whisker photocatalysts using photoreduction. Mater. Sci. Eng. B 176, 978–983. 10.1016/j.mseb.2011.05.027

[B45] Wei F. Liu L. Li G. (2014). Zinc oxide/copper oxide heterogeneous nanowire preparation and application in uv sensor, in 14th IEEE International Conference on Nanotechnology (Toronto, ON).

[B46] Wu M. Yan L. Li J. Wang L. (2017). Synthesis and photocatalytic performance of Ag/AgCl/ZnO tetrapod composites. Res. Chem. Int. 43, 6407–6419. 10.1007/s11164-017-2997-1

[B47] Yamakata A. Ishibashi T. Onishi H. (2002). Electron- and hole-capture reactions on pt/tio_2_ photocatalyst exposed to methanol vapor studied with time-resolved infrared absorption spectroscopy. J. Phys. Chem. B 106, 9122–9125. 10.1021/jp025993x

[B48] Yamakata A. Ishibashi T. Onishi H. (2003). Microsecond kinetics of photocatalytic oxidation on Pt/TiO_2_traced by vibrational spectroscopy. Chem. Phys. Lett. 376, 5–6. 10.1016/S0009-2614(03)01034-0

[B49] Zhang Q. Fan W. Gao L. (2007). Anatase TiO_2_ nanoparticles immobilized on ZnO tetrapods as a highly efficient and easily recyclable photocatalyst. Appl. Catal. B Environ. 76, 168–173. 10.1016/j.apcatb.2007.05.024

[B50] Zhao P. Feng X. Huang D. Yang G. Astruc D. (2015). Basic concepts and recent advances in nitrophenol reduction by gold- and other transition metal nanoparticles. Coord. Chem. Rev. 287, 114–136. 10.1016/j.ccr.2015.01.002

[B51] Zhu M. Sun Z. Fujitsuka M. Majima T. (2018). Z-Scheme photocatalytic water splitting on a 2D heterostructure of black phosphorous/bismuth vanadate using visible light. Angew. Chem. Int. Ed. 1, 2160–2164. 10.1002/anie.201711357 29276822

