# Supplementary material for: Light-Mediated Growth of Noble Metal Nanostructures (Au, Ag, Cu, Pt, Pd, Ru, Ir, Rh) From Micro- and Nanoscale ZnO Tetrapodal Backbones
Source: Front Chem. 2018 Sep 10;6:411. doi: 10.3389/fchem.2018.00411 (PMC6139342; doi:10.3389/fchem.2018.00411)
Supplement: Supplementary file 1 [file Data_Sheet_1.PDF]

Supplementary Material

Light-Mediated Growth of Noble Metal Nanostructures (Au, Ag, Cu, Pt, Pd, Ru, Ir, Rh) from Micro- and Nanoscale ZnO Tetrapodal Backbones

Trevor B. Demille[1], Robert A. Hughes[1], Arin S. Preston[1], Rainer Adelung[2], Yogendra Kumar Mishra[2] and Svetlana Neretina[1,3,4*]

[1] College of Engineering, University of Notre Dame, Notre Dame, Indiana, 46556, USA

[2] Functional Nanomaterials, Institute for Materials Science, Kiel University, Kaiserstra. 2, D-24143 Kiel, Germany

[3] Department of Chemistry and Biochemistry, University of Notre Dame, Notre Dame, Indiana, 46556, USA

[4] Center for Sustainable Energy at Notre Dame, Notre Dame, Indiana, 46556, USA

*** Correspondence:**
Svetlana Neretina
sneretina@nd.edu

1 Supplementary Figures

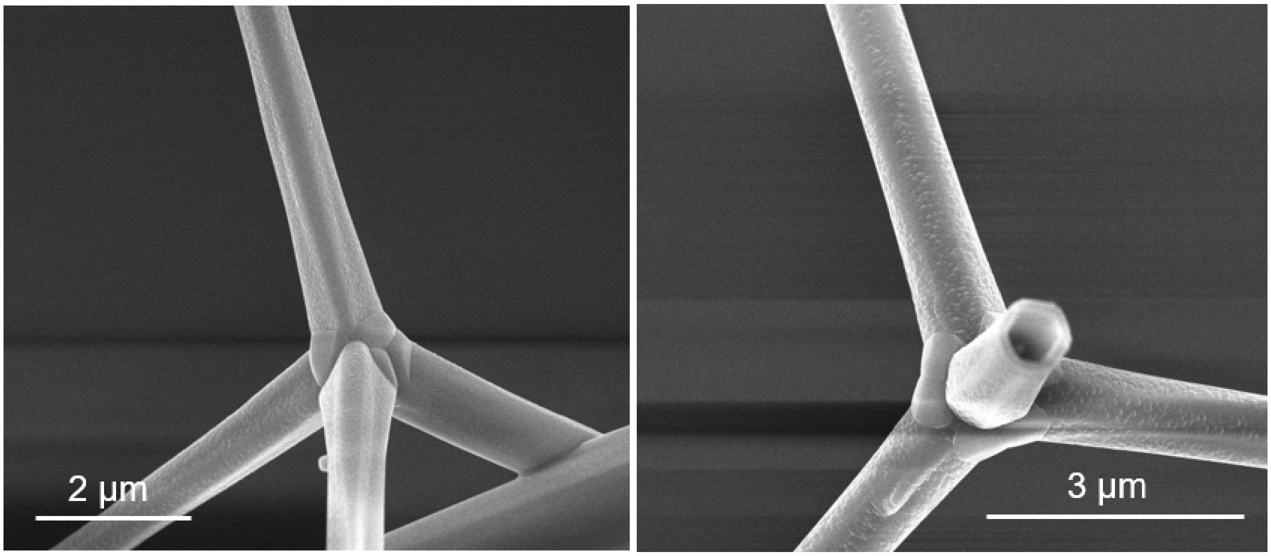

Supplementary Figure 1. SEM images of ZnO tetrapods decorated with Ir nanoparticles. It can be seen that Ir nanoparticle deposition has taken place preferentially on the tetrapod limbs (Wurtzite crystal structure) as opposed to the tetrapod cores (zincblende crystal structure).

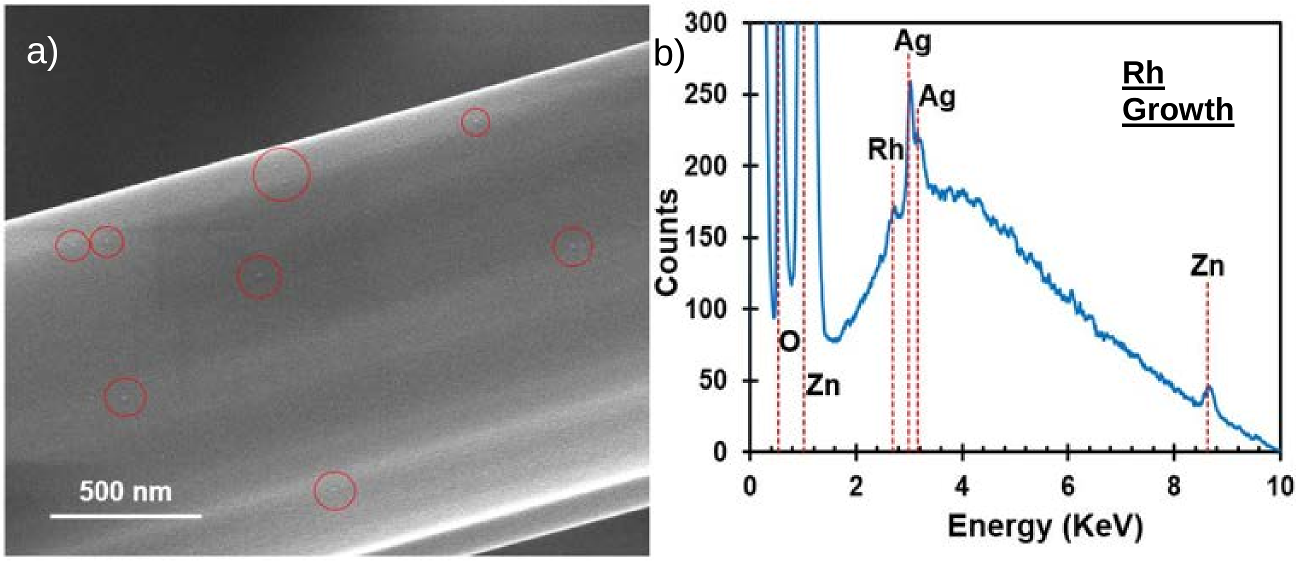

Supplementary Figure 2. a) SEM image of a ZnO tetrapod decorated with Rh nanoparticles where the nanoparticles are circled in red for clarity. It is likely that other Rh nanoparticles exist on the tetrapod that have diameters that are not readily resolvable in the SEM image. b) Energy dispersive X-ray spectroscopy (EDS) spectrum demonstrating the presence of a small Rh signature in addition to peaks associated with Zn, O, and Ag. The Ag peaks arise from the use of Ag paint as a means to affix the tetrapods to the SEM stub.

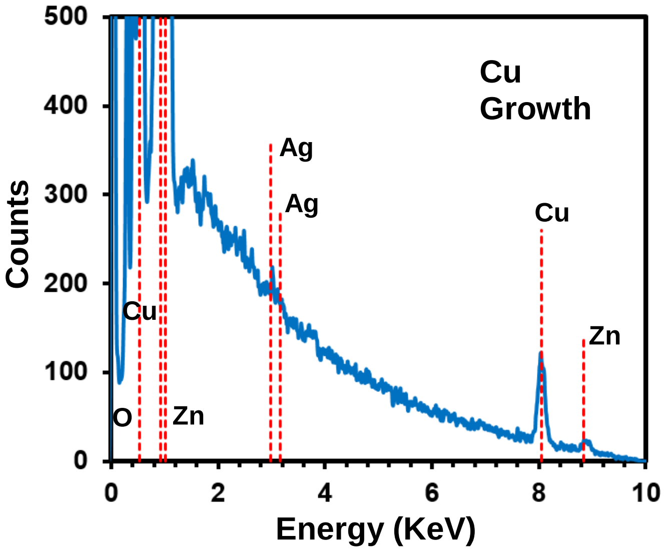

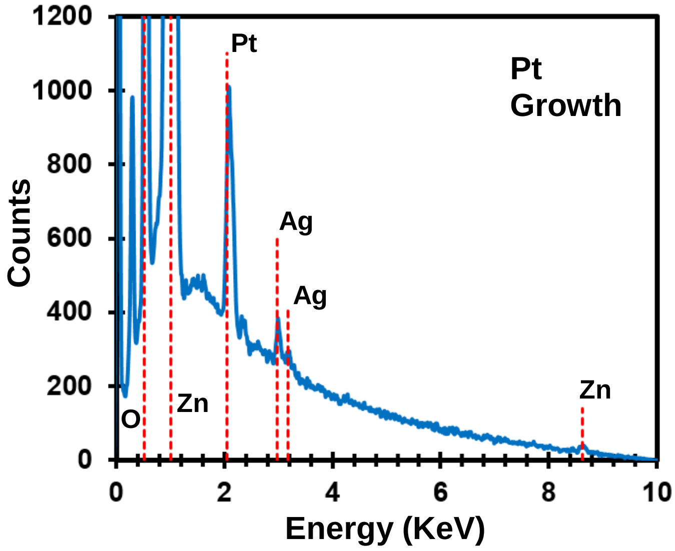

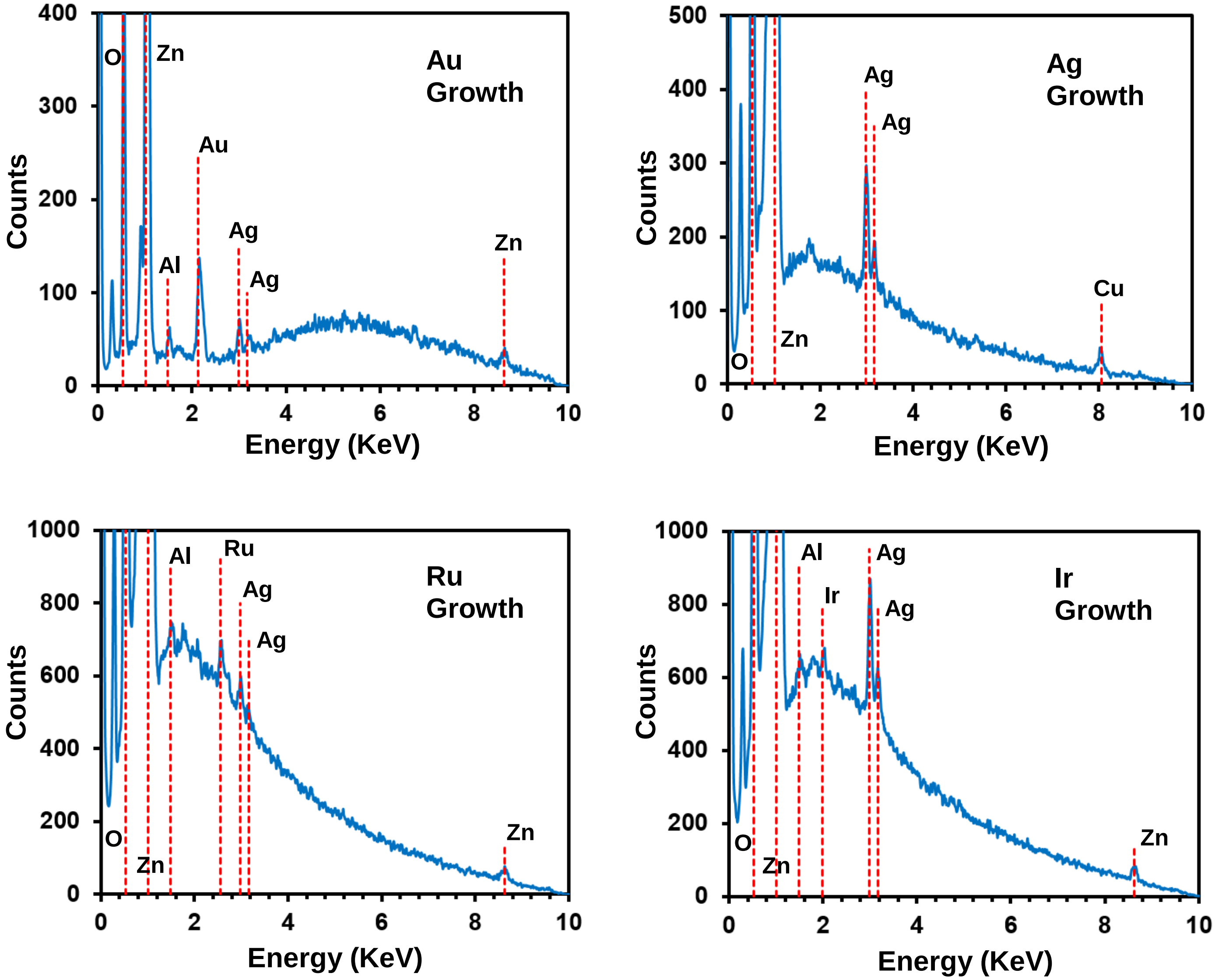

Supplementary Figure 3. Energy dispersive X-ray spectroscopy (EDS) data showing the Cu, Pt, Au, Ag, Ru, and Ir signatures in addition to Zn and O tetrapod peaks. The Ag and Al peaks arise from the use of Ag paint as a means to affix the tetrapods to the Al SEM stub. For the case of the Ag decorated tetrapods, Cu was used instead of Ag paint to affix the structures to the Al stub.
